# Vomiting of Pregnancy

**Published:** 1899-01

**Authors:** R. Brent Murphy

**Affiliations:** St. Louis


### VOMITING OF PREGNANCY.

By R. BRENT MURPHY, M.D., St. Louis.

Dr. Bacon in the *American Journal of the Medical Sciences* for June concludes as follows:

1. The abnormal irritability of the nervous system, including the vomiting center, is to be allayed by keeping the patient in the horizontal position; by attention to the skin, bowels, and kidneys; rectal and. if necessary, hypodermic injections of salt solution being used.
2. The hysterical condition, which is so commonly found present, should be controlled by strengthing the will and influencing the dominant ideas of the patient.
3. All sources of peripheral irritation should be discovered and treated.
4. In extreme cases subcutaneous saline injections serve a threefold purpose: (*a*) diluting the blood and increasing vascular tension; (*b*) eliminating toxines through renal and intestinal emunctories; (*c*) furnishing two most important kinds of food.
5. Induction of abortion is never indicated. At a stage when it is safe and efficient it is not necessary, and in extreme cases it adds greatly to the danger, rarely stops vomiting, and can be avoided by the use of artificial serum.

